# Do income inequality affect internet diffusion? Empirical evidence from night light data

**DOI:** 10.3389/fpubh.2025.1677208

**Published:** 2025-10-21

**Authors:** Jianshuang Fan, Yuanjia Wang, Sicheng Cai, Qiongfang Feng

**Affiliations:** ^1^The Chinese Academy of Housing and Real Estate, Zhejiang University of Technology, Hangzhou, China; ^2^School of Management, Zhejiang University of Technology, Hangzhou, China

**Keywords:** income inequality, internet diffusion, digital divide, night light data, China

## Abstract

**Background:**

Promoting the application of the Internet and information technology has become an inevitable choice if a country is to achieve the country’s goal of high-quality economic development. Rising income inequality may have a dampening effect on Internet diffusion and exacerbate the digital divide. However, the amount of literature on related issues is scant.

**Methods:**

This paper uses night light data to measure the Gini coefficients in China, and thereby to gauge the level of income inequality. Eventually, a panel data set covering 30 provinces and 272 prefecture-level cities in China from 2005 to 2020 is obtained.

**Results:**

Based on this data set, the baseline regression results suggest that income inequality significantly inhibits Internet diffusion. The threshold regression results suggest that with the improvement of regional economic level, as well as the level of residents’ social capital and human capital, the inhibitory effect of regional income inequality on Internet consumption is weakening. The results of the heterogeneity test show that the effects on Internet diffusion in the mid-western and non-innovative cities are stronger than in the eastern and innovative cities. The results of the mechanism test show that income inequality has a negative effect on Internet diffusion through the economic suppressive effect, education crowding-out effect, and class solidification effect.

**Conclusion:**

Income inequality significantly inhibits Internet diffusion. This paper provides theoretical insights and decision-making references for effectively promoting Internet diffusion from the perspective of income inequality.

## 1 Introduction

In the 21st century, information and communication technologies have continuously developed. Both the breadth and depth of Internet diffusion have been constantly strengthened. With the advent of the digital economy, the Internet has also become increasingly important. Internet technology has penetrated all aspects of work and daily life and has had a broad and profound impact on the national economy, society, politics, and government (1). The Internet can improve market efficiency, save production and operating costs, create new employment opportunities, and give rise to new industries and new forms of business (2). Computer literacy is positively correlated with social engagement and employment prospects (3–5). However, while promoting socio-economic development, Internet technology has also given rise to new inequalities and social divisions. Specifically, this is known as the digital divide (6). The digital divide, which is caused by differences in the ownership, skills, and applications of the Internet has led to other social inequalities. Examples include a widening of the wealth gap, significantly reduced total household income, weakened social networks, inhibited entrepreneurship, and reduced credit availability (3, 7, 8). This is because Internet technology has skill bias characteristics that enable groups with information processing advantages to gain economic benefits and thereby widen income gaps between different groups (5).

**Figure 1:**
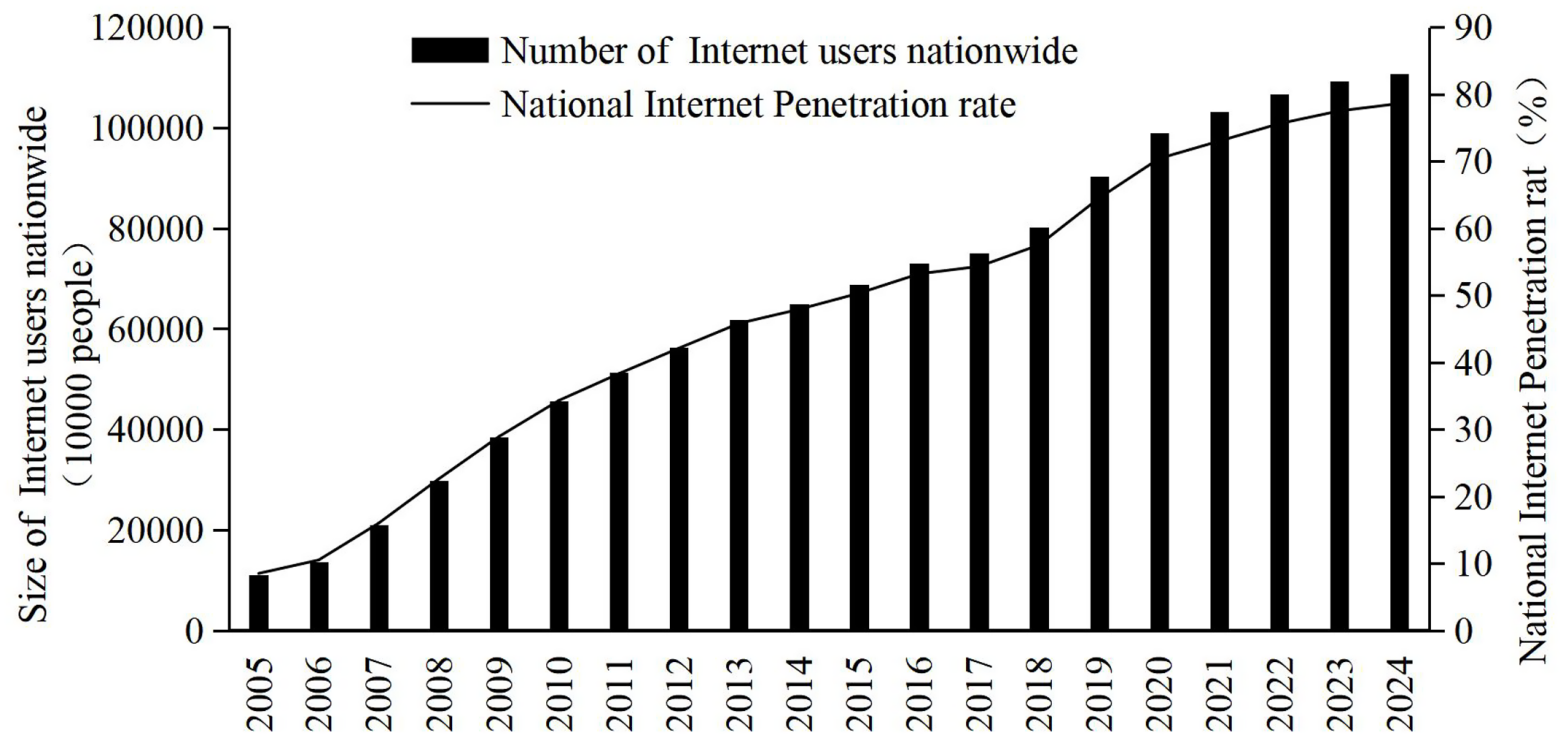
Trends in the number of internet users nationwide and the penetration rate. Source: statistical report on internet development in China.

The Chinese government attaches great importance to the promotion and application of the Internet. As such, the strategic objectives of building an “Internet powerhouse” and a “digital China” have been proposed. By the end of 2024, the number of Internet users in China had reached 1.108 billion, or 15.76 million more than that in 2023. The Internet penetration rate had reached 78.6%.^1^ However, internet penetration is approaching saturation in countries including Denmark, the Netherlands, Norway, Saudi Arabia, Switzerland, and the United Arab Emirates. In order to alleviate certain problems, such as the relatively lagging Internet diffusion rate in China’s central and western regions, and to consistently narrow the Internet diffusion gap among regions, the Chinese government introduced several successive policies and measures. One example is the “broadband China” special action plan, launched in 2014. During this period, China’s overall Internet penetration rate and the number of Internet users increased dramatically, from 8.5% and 110 million in 2005, to 78.6% and 1.108 billion in 2024, respectively. The Internet itself has also experienced explosive growth (Figure 1). The Chinese government has introduced numerous policies and measures to promote Internet diffusion. However, the urban–rural gap in Internet diffusion still exists. By the conclusion of 2024, the Internet penetration rate among urban residents in China reached 85.3%, yet for rural residents, it was merely 67.4%. This significant disparity clearly indicates that the digital divide between urban and rural areas persists. The diffusion of the Internet is intricately influenced by a confluence of social, economic, political, cultural, and technological factors. As a result, the causes underlying the digital divide are multifaceted and complex (9). Given this situation, there is an urgent imperative to conduct in-depth research on the primary factors influencing Internet diffusion.

Therefore, promoting Internet diffusion through the improvement of residents’ incomes is not just an important measure to narrow the digital divide. This is also an important starting point for China to promote the development of the digital economy. Improved incomes will play a decisive role in China’s realization of the goal of building a “digital China,” promoting China’s path to modernization, and promoting the deep integration of the digital and the real economy. Based on the market supply and demand theory, the diffusion of the Internet is predominantly influenced by two key factors: the cost of Internet access and the income levels of residents (10). Consequently, the primary driving forces for promoting Internet diffusion are the steady elevation of residents’ income levels and the consistent decrease in Internet access costs. Achieving the latter depends significantly on advancements in Internet technology (10). With higher incomes, more people can afford Internet services. Simultaneously, as Internet technology progresses, infrastructure becomes more efficient, competition increases, and ultimately, the cost of providing Internet access drops. This dual-pronged approach (raising incomes and reducing costs) is essential for broader and more rapid Internet diffusion.

However, the presence of income inequality has emerged as a pivotal hurdle for China in its pursuit of the “Digital China” objectives and the widespread diffusion of the Internet. According to data from the World Bank, China’s Gini coefficient in 2022 stood at 0.474, far exceeding that of numerous developing and developed nations. Rising income inequality exerts detrimental effects on various aspects, most notably consumer demand and human capital accumulation. It has also morphed into a critical factor that not only impedes the spread of the Internet but also exacerbates the digital divide. Consequently, conducting in-depth research into the impact of income inequality on Internet diffusion holds immense theoretical value and practical significance, which can offer valuable insights for formulating policies to narrow the digital divide, promote more inclusive Internet development, and ensure that the benefits of the digital age are more evenly distributed across society.

Given the significant impact of income inequality on the social economy (11), income inequality and consumption have emerged as topics garnering extensive attention across academia, politics, and society. Scholarly research has revealed that income inequality spurs families to boost investment in human and social capital, and increase precautionary savings. Concurrently, it crowds out consumption of commodities for survival and enjoyment (12). Higher inequality exacerbates credit constraints for lower-income households, raising the cost of financial market entry and suppressing consumption (13). Bricker et al. (14) discovered that in counties with greater inequality, relatively well-off households’ resort to more debt to buy cars and other tangible goods. They do this to flaunt their higher income status, aiming to penetrate lucrative social networks. Regarding overall social consumption, income inequality mainly curbs the consumption level of the poor, resulting in insufficient overall social consumption capacity (15). Concurrently, inequality reinforces socio-psychological disparities. It amplifies status hierarchies, which triggers negative psychosocial responses such as stress and reduced social capital, while also fostering status-seeking and conspicuous consumption as individuals respond to perceived inferiority (16, 55). Furthermore, inequality erodes social trust by widening gaps in life satisfaction (17).

Notably, much of the existing literature on the implications of income inequality has centered on the consumption perspective. However, studies zeroing in on the impact of income inequality on Internet diffusion are remarkably scarce. At the macro level, income inequality can curb economic growth. Slow economic growth means less funding and incentives for Internet infrastructure construction. Since Internet infrastructure is essential for better speed, stability, and security, as well as for wide-scale Internet diffusion, this is a significant issue. At the micro level, income inequality dampens consumption, human capital, and social capital accumulation. These factors are crucial for Internet diffusion. As a result, the negative impact of income inequality on Internet diffusion has become a key obstacle to the healthy and sustainable development of the Internet and the information and communication technology (ICT) industry.

Therefore, we need to clarify how income inequality affects Internet diffusion and its underlying mechanisms. This will offer decision-making references for the rapid development of China’s ICT and digital economy sectors. This paper specifically explores whether and how income inequality in China has an impact on Internet diffusion. Specifically, the Gini coefficient measured by nighttime lighting data is adopted to accurately identify the level of income inequality at the provincial and municipal levels in China, respectively. Then an empirical test is conducted on the effects and intermediate mechanisms of income inequality on Internet diffusion. This study also empirically tests whether the impact of income inequality on Internet consumption has potential nonlinear and heterogeneous characteristics.

The potential marginal contributions of this paper are mainly manifested in two key aspects. First, we expand the scope of the literature on the impact of income inequality. While most existing studies concentrate on the influence of income inequality on consumption, we are the first to identify the causal link between income inequality and Internet diffusion in the Chinese context. We further explore the nonlinear effects and heterogeneity characteristics of this relationship, aiming to provide practical experience and decision-making references for promoting Internet diffusion through reducing income inequality. Second, we make contributions in terms of constructing influence paths. We systematically build the paths through which income inequality impacts Internet diffusion from three aspects: the economic suppressive effect, education crowding-out effect, and class solidification effect, enriching the existing literature. Moreover, we innovatively use night light data and the Gini coefficient to scientifically measure income inequality, as these methods can better reflect the actual situation, thus offering a new methodological perspective for future research.

The remainder of this paper is organized as follows. Section 2 presents the literature review and theoretical framework. Section 3 details the data sources and the research methods employed. Section 4 showcases the empirical results. Section 5 delves into the results of mechanisms analysis. Section 6 concludes this paper and presents the corresponding policy implications.

## 2 Literature review and theoretical framework

### 2.1 Determinants of internet diffusion

Internet diffusion has obvious network effects (18). Effectively reducing the Internet use cost and continuously lowering the Internet use threshold are particularly important for increasing Internet diffusion level in China, thus better promoting the development of digital economy. In this regard, the literature has explored the influencing factors of Internet diffusion from the perspectives of education level, Internet infrastructure, institutional quality, income level and Internet access cost. In terms of educational attainment, studies have found that increased educational attainment is an important driver of Internet diffusion (10, 19). People with high incomes and good education are more likely to access Internet and have a higher likelihood of demand for advanced Internet products and services (20–22). Disparities in education within countries and regions can have a dampening effect on Internet diffusion (7). High education level can have a positive impact on Internet diffusion. Conversely, people with low education level have difficulty acquiring the equipment and skills to use Internet, and therefore the employment opportunities offered by the digital labor market (6, 23).

In terms of Internet infrastructure, it is an important factor influencing Internet diffusion (1, 24). Li and Shiu (18) found that educational attainment and Internet infrastructure development are important factors affecting Internet diffusion. In terms of institutional quality, market regulation and government behaviors are important factors influencing Internet diffusion (25). High levels of national and regional government regulation can lead to better Internet diffusion (26). High institutional quality can accelerate the diffusion of Internet, i.e., broad democracy is conducive to narrowing the digital divide (1, 9, 27). The reason is that democracy has innovative advantages (28).

In terms of income level of residents and Internet access cost, some studies have found that the income level is the most important determinant of increasing Internet diffusion (1, 18, 29). Hargittai (30) found that residents’ income and Internet policies are the most important factors affecting Internet diffusion. Kiiski and Pohjola (10) suggested that Internet diffusion is influenced by both price and income. That is, by reducing the cost of Internet access and increasing the income of residents are important ways to increase Internet diffusion. Since different Internet providers offer Internet services at different prices, consumers will switch between providers after considering the differences in Internet diffusion costs (31). In addition to the cost of Internet access, Internet content and services are also important factors affecting Internet diffusion (32, 33).

In addition to the above influences, entrepreneurship and geographic location are also important factors affecting Internet diffusion (2, 34–36). However, existing literature mainly uses cross-country data as the research object. Currently, there is scarce literature specifically centered on regional-level data in China. Moreover, even fewer studies utilize both provincial and prefectural-level data to examine the impact of income inequality on Internet diffusion. This presents an opportunity for the marginal contributions of this paper.

### 2.2 Theoretical framework

Internet diffusion is a commercialization process. Therefore, the micro-mechanisms of Internet diffusion can be explained by innovation diffusion theory, technology acceptance model, and consumption theory. Among them, innovation diffusion theory was first proposed by Rogers (37). The theory breaks down the process of innovation diffusion into the five stages of acquisition, persuasion, decision-making, implementation, and confirmation. The process of innovation diffusion is also considered to include the four elements of innovation, diffusion channel, time, and social system. Innovation diffusion theory also proposes a core indicator, namely the rate of innovation adoption, i.e., the relative speed of innovation adoption by members of the social system. The focus on the Internet is to determine the number of society members who become Internet users over a given period. Notably, several studies have previously confirmed that the ICT and Internet adoption process basically conforms to the S-curve trend (38–40). The changes in the curve are characterized by a slow start in the adoption rate of innovation, followed by a rapid increase in the adoption rate as the number of innovation adopters increases.

The technology acceptance model proposed by Davis (41) and Davis et al. (42) also represents a theory that explains technology diffusion. The model is based on reasoned behavior theory and planned behavior theory and proposes two determinants of technology diffusion. The first determinant is perceived usefulness, which reflects the extent to which individuals believe that using a specific technology enhances their job performance. The second determinant is perceived ease of use; this reflects the degree to which individuals find it easy to use a specific technology. The model mainly emphasizes the impact of individual attitudes and willingness on technology diffusion. After the initial study, the model was further expanded to state that individual willingness and technological diffusion are composed of the four core elements of performance expectations, effort expectations, social influence, and convenience conditions (43).

Consumption theory emphasizes how consumers make purchasing decisions and argues that consumers will maximize utility within their income budget constraints, in order to make rational choices. The equilibrium point of consumption lies in an individual’s maximization of utility at his or her budget threshold (44). Different income levels correspond to different consumer equilibrium points.

**Figure 2:**
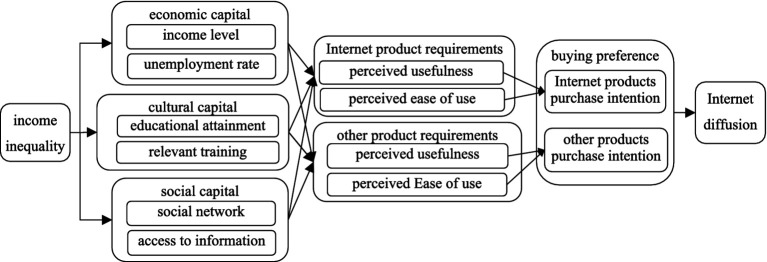
Conceptual model.

According to Bourdieu’s theory of capital, it is classified into three primary forms: economic, cultural, and social capital. This typology offers a robust analytical framework for research. Drawing on these theoretical foundations, along with insights from Zhang (44), we develop a comprehensive conceptual model, as illustrated in Figure 2.

The process of Internet diffusion is not solely a technical issue but also a social issue, closely related to many inequalities in socio-economic development. For example, whether consumers can participate in Internet use, acquire Internet use skills, and benefit from Internet use are complex social issues (9). Based on established theoretical foundation and the findings of previous literature, this paper argues that the impact of income inequality on Internet diffusion includes both direct and indirect effects.

#### 2.2.1 The direct impact mechanism

The degree of Internet diffusion is primarily determined by purchase preference and ability to pay two factors. Residents use the Internet mainly for four purposes: social activities, entertainment, information gathering, and business transactions. The concept of “purchase preference” reflects the possible order of consumers’ preferences for Internet products and other goods. Moreover, purchase preference depends on the perceived usefulness and perceived ease of use of the products. The ability to pay is determined by the price of Internet products and consumers’ income levels.

First, the widening income gap concentrates wealth among a select few, increasing the number of individuals with lower incomes and higher rates of joblessness. If the cost of Internet access remains constant, the growing population of low- and middle-income groups, who constitute the majority, will cause a general decrease in Internet usage across society. Although high-income groups possess sufficient payment capacity, their marginal propensity to consume is significantly lower than that of low-income groups. Thus, their willingness to increase consumption is weak (45). Conversely, low-income groups may have a latent demand for Internet products but are unable to convert it into actual consumption due to limited income. Since the demand for Internet services among the wealthy minority is limited, most of the existing social wealth does not flow into the Internet market.

Second, while Internet diffusion theoretically covers all demographic groups, the weak consumption willingness among low- and middle-income groups remains a major obstacle to Internet diffusion. For low- and middle-income groups, the perceived usefulness and ease of use of Internet products are relatively low. They face high costs for trial and error, harbor distrust toward the online consumption environment and their own capabilities (19), and they lack the preference and willingness to purchase Internet services, which restricts their Internet-related consumption (8). Furthermore, low-income groups tend to limit their Internet usage to basic, low-cost entertainment or social functions, rather than engaging in productive activities that could yield higher economic returns (32, 33). As a result, their Internet usage use fails to translate into tangible benefits, further reducing their willingness to use Internet products.

From a socio-psychological perspective, when individuals perceive a significant disparity between their economic status and that of higher groups, they are prone to experiencing relative deprivation and status anxiety. Intense relative deprivation undermines consumption confidence, particularly suppressing the desire to purchase non-essential goods (Pybus et al., 2024). Low-income groups may opt to curtail spending and increase savings to cope with uncertainty, thereby lowering their overall marginal propensity to consume (12). Expenses related to Internet access and services may be deemed non-urgent or reducible, especially when basic living needs are under pressure. The long-term benefits of Internet access, such as information acquisition, online education, and skill enhancement (46), may give way to more pressing short-term survival needs (4, 47). Conversely, narrowing the income gap can boost Internet diffusion (2), enhance the network value of products, and thereby drive innovation and upgrading in the Internet industry. The first hypothesis (H1) to be tested of our paper is:

H1: Income inequality has a negative impact on Internet diffusion.

#### 2.2.2 The intermediate impact mechanism

The intermediate mechanisms through which income inequality impacts Internet diffusion incorporate both macro and micro aspects. Macroscopically, local economic development, which underpin Internet consumption, is closely tied to Internet consumption levels. However, growing income inequality impedes economic growth. This, in turn, obstructs large-scale information infrastructure construction. Microscopically, income inequality deteriorates residents’ cultural and social capital levels. This is harmful to Internet access and the enhancement of Internet skills among current and potential users, thereby impeding Internet diffusion.

The *Guiding Opinions on Accelerating the Construction of High-speed Broadband Networks and Promoting Network Speed Increase and Fee Reduction* was issued by the General Office of the State Council of China. The document pointed out that the upgrading and reconstruction of broadband infrastructure in areas with weak infrastructure should be supported in combination with national strategies. Such strategies include new urbanization, the “the Belt and Road,” and the Yangtze River Economic Belt. Therefore, to a certain extent, the level of economic growth determines the level of investment in Internet infrastructure, which in turn determines the level of Internet diffusion (1, 24). Weak economic growth limits the investment level of Internet infrastructure and thus discourages Internet diffusion. This study defines this mechanism as the “economic suppressive effect” of income inequality.

Human capital is an important factor that influences Internet diffusion. Education is the most important means to promote Internet consumption, because only through education, training, and continuous learning can people master digital technology and capabilities. Education is the avenue through which people become interested in Internet consumption and applications (10, 19). Income inequality contributes to the expansion of the low- and middle-income groups. This expansion, in turn, exacerbates disparities in the distribution of educational resources, diminishes general access to education, and impedes the ability of low-income groups to invest in human capital. Low-income groups have very limited access to the Internet; they are also unwilling or unable to pay Internet technology training fees. This results in a lack of ability to use Internet technology, which significantly reduces residents’ perceived usefulness and perceived ease of use regarding the Internet. Higher-income groups usually have good education, and high skill levels (1). This makes accessing the Internet easier for these groups. As a result, in areas with high income inequality, Internet diffusion is relatively, low due to the low mobility, low educational attainment, and lack of Internet skills training of the poorer groups (2, 47). This study refers to this mechanism as the “educational crowding-out effect” of income inequality.

An increase in social capital can significantly improve residents’ resource allocation ability and employment opportunities, which in turn translates into the ability to acquire income. In addition, income is an important factor in determining the Internet diffusion level (18, 29). Unfortunately, income inequality weakens residents’ social capital. The nodes and quality of the social networks of low- and middle-income groups are poor, making it difficult for these groups to obtain useful resources and information related to Internet technology. It is also impossible for them to obtain and understand effective Internet information and that information’s application value. This significantly reduces the perceived usefulness of Internet products for these groups and then reduces their willingness to pay and their purchasing behavior. Ultimately, the Internet diffusion level will also be reduced. This study refers to this mechanism as the “class solidification effect” of income inequality. The second hypotheses (H2) to be tested in this paper is:

H2: Income inequality has a negative effect on Internet diffusion through the economic suppressive effect, education crowding-out effect, and class solidification effect.

## 3 Data and method

### 3.1 Empirical strategy

#### 3.1.1 Baseline regression model

To explore the impact of income inequality on Internet diffusion, this paper designs the following baseline regression model:


(1) $$\mathrm{InIu}s_{i,t}=\alpha_{0}+\alpha_{1}Ig_{i,t}+\lambda_{j}\sum_{j-1}^{J}X_{i,j,t}+u_{i}+v_{t}+\varepsilon_{i,t}$$

Where *i* denotes a province or city, and *t* denotes the year; ln*Ius_i,t_* is the dependent variable, which denotes the Internet diffusion level; *Ig_i,t_* is the core independent variable, which denotes income inequality, and *X_i,t_* denotes a set of control variables. Then, *μ_i_* denotes the province or city fixed effect, *v_t_* denotes the year fixed effect, and *ε_i,t_* denotes the random error term. The model standard errors are also corrected for robustness.

#### 3.1.2 Panel threshold model

Income inequality may exert a non-linear impact on Internet diffusion. Meanwhile, this impact can be influenced by the degree of income inequality, as well as mediating variables such as economic development, human capital, and social capital. Based on this, we further construct the following panel threshold model:


(2) $$\begin{array}{l} \ln Ius_{i,t}=\phi_{0}+\phi_{0}Ig_{i,t}\times I\left(Adj\leq \eta_{1}\right)+\phi_{2}Ig_{i,t}\times I\left(Adj>\eta_{1}\right)\\ \;+\lambda_{j}\sum_{j=1}^{J}X_{i,j,t}+u_{i}+v_{t}+\varepsilon_{i,t} \end{array}$$

Where *Adj_i,t_* denotes the five threshold variables of income inequality, economic development, human capital, and social capital; *η*_1_ denotes the threshold value, and *I*(·) is the indicator function that takes the value of 1 if it meets the conditions in the parentheses and 0 otherwise. Equation 2 considers the single-threshold scenario. The equation can also be expanded to the multi-threshold scenario, based on the econometric test of the sample data and other steps.

### 3.2 Data selection

#### 3.2.1 Dependent variable

The dependent variable is the Internet diffusion rate (ln*Ius*). We use the number of Internet users per 100 people to measure ln*Ius* for the province sample (32). As only a small fraction of internet users is under 15, we use the population over 15 to represent the total population. The calculation formula is as follows: Internet diffusion Rate = number of internet users ÷ (population over 15 years of age in the sample survey ÷ total population in the sample survey × number of permanent residents at the end of the year) × 100. The relevant data come from the population sample survey published by *China Internet Network Information Center and Statistical Yearbook*.

Drawing on the research of Beilock and Dimitrova (1), we use the number of permanent residents at the end of the year to characterize the total population for the city sample, and we calculate ln*Ius* as follows: Internet diffusion rate = the number of Internet broadband access users ÷ the number of permanent residents at the end of the year × 100.

We also introduce Internet user size (ln*Int*) and per capita telecom traffic volume (*Ttv*) as alternative indicators of ln*Ius* for robustness testing. Given the wide disparities in Internet diffusion rates and user sizes among cities, we log-transform these two variables in the empirical test. This mitigates data volatility, rendering the analyzed data more stable.

#### 3.2.2 Independent variable

The independent variable is income inequality (*Ig*). We use the nighttime light data from the DMSP-OLS and NPP-VIIRS satellites of the NOAA of US to measure *Ig* for the province sample (5). These data act as proxies for the economic development of each district and county because the intensity of nighttime light radiation can reflect the activity levels of residents. By applying the Gini coefficient method to the nighttime light luminance differences across districts and counties within each province, we can characterize provincial income inequality (2, 12).

For robustness testing, multiple alternative approaches are employed. First, the average nighttime light brightness of each city is adopted as a proxy for city-level economic development, and the Gini coefficient (*Ig_G*) is calculated for the differences in nighttime light brightness among cities within a province, providing a replacement variable for provincial income inequality. Municipalities, which are directly divided into districts and counties without city-level data, are excluded from relevant results. Second, the Gini coefficient based on the per capita GDP of cities nationwide *(Ig_L*) serves as another replacement variable for income inequality. Moreover, to measure income inequality for the city sample, the same method used for provinces is applied, calculating the Gini coefficient of the mean nighttime light brightness of districts and counties within each city.

#### 3.2.3 Intermediate variables

##### 3.2.3.1 Economic development (ln*GDP*)

We use the per capita regional gross domestic product of residents to measure GDP and deflate it through the GDP index (10, 32). The higher the economic development level, the more funds it can invest in Internet technology applications and Internet infrastructure construction (2).

##### 3.2.3.2 Human capital (*Huc*)

We use years of education per capita to measure *Huc* for the province sample (10). The formula for calculating *Huc* is as follows: *Huc* = (number of illiterate people × 1 + number of people with elementary school education × 6 + number of people with middle school education × 9 + number of people with senior high school and middle school education×12 + number of people with college and bachelor’s degree or higher education×16) ÷ total number of people over 6 years old. The relevant data is from the China Statistical Yearbook and the Seventh Population Census. We use the teacher-student ratio to measure *Huc* for the city sample. The formula is as follows: *Huc* = number of full-time teachers in general secondary and elementary school ÷ number of students enrolled in general secondary and elementary school.

#### 3.2.4 Social capital (*Soc*)

In this study, social capital constitutes a normative social network relationship predicated on empathy, trust, and reciprocity. Non-Governmental Organizations (NGOs) generally refer to societal organizations independent of the government, which explicitly exclude profit-seeking enterprises and other similar entities. These organizations are predominantly engaged in social public welfare activities, representing the reciprocal and trust-based relationships among people within a community. NGOs characterized by their public welfare orientation, openness, and fairness, serve as a reflection of a region’s level of mutual understanding, trust, and care, thereby embodying the extent of local empathy and reciprocity (48). Therefore, the number of NGOs per capita is used to measure social capital, under the assumption that it reflects the vibrancy of associational life conducive to normative social networks. These data are directly obtained from *the China Civil Affairs Statistical Yearbook*.

#### 3.2.5 Control variables

##### 3.2.5.1 Entrepreneurship level (ln*Ent*)

We use the number of new business registrations in the year to measure entrepreneurship level which are obtained from *the China’s State Administration for Market Regulation*. The database contains the whole national industrial and commercial firm registration data in China, covering over 250 million new registered firms. Entrepreneurship fosters technological and business model innovation by intensifying market competition, compelling enterprises to enhance service quality and reduce costs (35). This dynamic creates favorable conditions for the widespread adoption and deeper application of Internet technologies, thereby facilitating their integration and innovation across various economic and social sectors.

##### 3.2.5.2 Marketization level (*Mar*)

We use the marketization index to measure *Mar*, with the calculation method following the idea of Wang et al. (49). A higher marketization level offer residents with better and cheaper Internet services, thus facilitating Internet diffusion (2).

##### 3.2.5.3 Age structure (*As*)

We use the percentage of population aged 15 to 64 to measure *As* for the province sample (7). From an age-preference perspective, young people are more receptive to new knowledge and technology. Most of today’s youth started using the Internet during their school years, making them more familiar with and heavy users of it (20). In contrast, the older adult(s) has declining cognitive abilities and relatively poor IT skills. Their long-standing habits also result in low interest and demand for Internet use (4). Thus, areas with a higher proportion of young people show greater Internet-using willingness (50). We use the natural population growth rate to measure *As* for the city sample.

##### 3.2.5.4 Informatization level (*Ifm*)

We measure *Ifm* by the proportion of total postal and telecommunications business in regional GDP (10). A region’s strong reliance on the digital economy or a high level of industrial digitization can boost more extensive and frequent Internet use (7).

##### 3.2.5.5 Transportation infrastructure level (*lnRoa*)

We measure it using per capita road area. In regions with well-developed transportation, accessing express delivery and takeout is more convenient. Also, those with a strong online shopping demand are more likely to use the Internet. This improves intra-regional connectivity, thus promoting Internet diffusion.

##### 3.2.5.6 Internet infrastructure level (ln*Iil*)

We use the per-capita length of fiber optic cable lines to measure it for the province sample (10). Expanding fiber optic cable length, increasing cell phone base stations, and investing in IT-related industries boost the supply of Internet technology and products (2). In a relatively stable-demand market, more network infrastructure supply can enhance Internet diffusion (32, 34). Due to data unavailability, a smart-city-pilot dummy variable is introduced to measure ln*Iil* for the city sample.

##### 3.2.5.7 Unemployment (ln*Une*)

We use the number of registered unemployed people in urban areas to measure it. Workers’ intrinsic motivation to acquire digital skills for enhanced work efficiency, market information access, and expanded business opportunities, in turn, accelerates the penetration and widespread adoption of internet technology in both production and daily life (51). Financially constrained, unemployed individuals often lack the means to afford Internet services (50).

##### 3.2.5.8 Institutional quality (*Cri*)

A high-quality and inclusive institutional environment is vital for Internet diffusion (2, 7, 26, 32). We use the crime rate to measure *Cri* for the province sample. The crime rate is quantified by the incidence of criminal cases in Chinese provinces. We use the degree of local government policy intervention to measure *Cri* for the city sample. The formula is: Local government policy intervention = (Fiscal expenditure - Science and education expenditure) ÷ Regional GDP.

### 3.3 Data sources and descriptive statistics

Given data availability, we use as research samples the panel data of 30 Chinese provinces (excluding Tibet due to data missing) and 272 prefectural-level cities from 2005 to 2020. The data is from the EPS data platform of the State Intellectual Property Office (SIPO), Chinese Research Data Services, CNRDS, China Urban Statistical Yearbook, China Statistical Yearbook, and China Population Census Yearbook. Table 1 reports the descriptive statistics. For the province sample, the minimum value of ln*Ius* is merely 1.077, while the maximum value reaches 230.957, a staggering 214-fold difference. This stark contrast clearly indicates a substantial gap in Internet diffusion among provinces. Regarding *Ig*, its mean value stands at 0.515, with a maximum of 0.784 and a minimum of 0.047. The maximum value is 16.7 times the minimum, once more highlighting the significant variation in income inequality levels across different provinces. Moreover, marked disparities are also evident in the values of mediating variables and control variables.

**Table 1:** Descriptive statistics of variables.

Sample type	Variables	Variable abbreviations	Observed value	Mean	Standard deviation	Min	Max
Province sample	Internet diffusion (Persons/100persons)	ln*Ius*	480	50.877	27.162	1.077	230.957
	Income inequality	*Ig*	480	0.515	0.159	0.047	0.784
	Economic development (10,000 yuan)	ln*GDP*	480	1.200	0.771	0.326	4.712
	social capital (per 10,000 persons)	*Soc*	480	4.349	1.954	1.220	12.021
	human capital (year)	*Huc*	480	8.880	1.042	6.378	12.782
	Entrepreneurship (number)	ln*Ent*	480	41.659	42.979	0.642	376.365
	Marketization	*Mar*	480	7.581	1.835	3.359	11.934
	Age structure (%)	*As*	480	0.731	0.037	0.635	0.838
	Informatization (%)	*Ifm*	480	0.067	0.046	0.014	0.290
	Transportation infrastructure (m^2^)	ln*Roa*	480	14.275	4.791	4.040	26.780
	Internet infrastructure (Kilometers per 10,000 persons)	ln*Iil*	480	161.794	124.632	19.294	640.291
	Unemployment (10,000 persons)	ln*Une*	480	25.576	13.951	2.900	73.900
	Institutional quality (%)	*Cri*	480	0.059	0.028	0.010	0.129
	Internet user size (10,000 persons)	ln*Int*	480	1846.092	1634.185	29.000	10923.000
	Telecommunications services per capita (10,000 yuan)	*Ttv*	480	0.263	0.280	0.042	1.484
	income inequality I	*Ig_G*	416	0.247	0.084	0.010	0.493
	income inequality II	*Ig_L*	416	0.417	0.152	0.085	0.782
City sample	Internet diffusion (Households/100 persons)	ln*Ius*	4,352	16.314	13.270	0.089	130.401
	Income inequality	*Ig*	4,352	0.566	0.212	0.001	0.958
	Economic development (10,000 yuan)	ln*GDP*	4,352	4.196	3.164	0.240	21.549
	social capital (per 10,000 persons)	*Soc*	4,352	4.255	1.933	1.220	12.021
	Human capital (%)	*Huc*	4,352	6.667	1.376	1.721	14.410
	Entrepreneurship (number)	ln*Ent*	4,352	4.402	6.120	0.064	90.440
	Marketization	*Mar*	4,352	10.381	2.865	2.717	19.694
	Age structure (%)	*Ps*	4,352	5.675	5.212	−16.640	40.780
	Informatization (%)	*Ifm*	4,352	0.027	0.020	0.000	0.274
	Transportation infrastructure (m^2^)	ln*Roa*	4,352	15.675	7.158	1.370	60.070
	Internet infrastructure (Kilometers per 10,000 persons)	ln*Iil*	4,352	0.169	0.375	0.000	1.000
	Unemployment (10,000 persons)	ln*Une*	4,352	2.561	2.979	0.077	31.614
	Institutional quality (%)	*Cri*	4,352	14.569	8.519	2.462	91.094
	Internet user size (10,000 Households)	ln*Int*	4,352	77.836	108.346	0.286	1424.10
	Telecommunications services per capita (10,000 yuan)	*Ttv*	4,352	0.108	0.165	0.001	1.650

## 4 Empirical results

### 4.1 Characteristic description of the relationship between two variables

**Figure 3:**
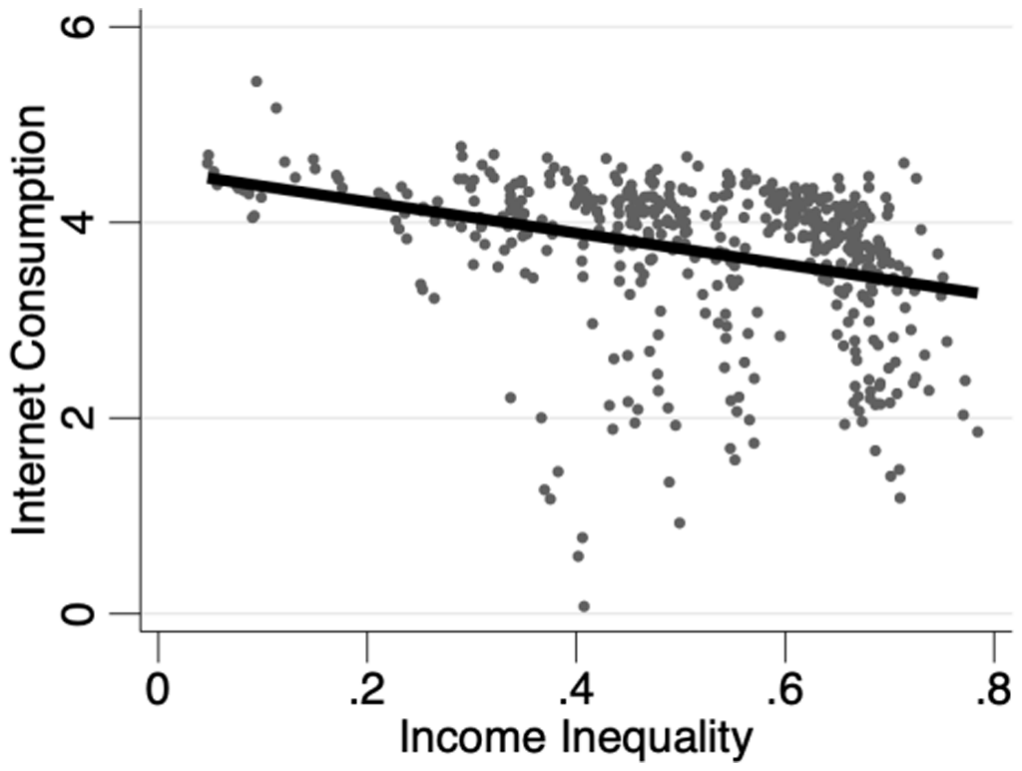
Scatterplot of province sample.

**Figure 4:**
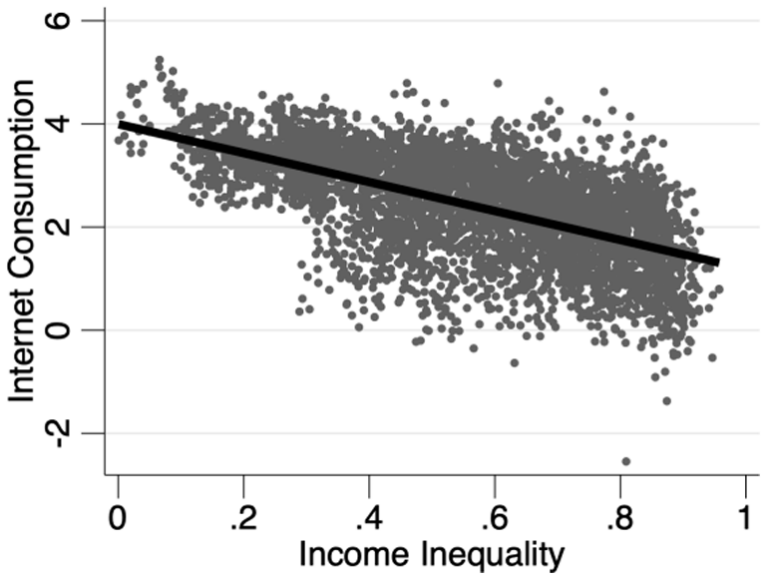
Scatterplot of city sample.

To better elucidate the correlation between income inequality and Internet diffusion, we plot scatter diagrams of income inequality against Internet diffusion. Figure 3 is based on province sample, while Figure 4 uses city sample. Evidently, provinces or cities with lower income inequality exhibit relatively higher Internet diffusion levels. This preliminarily validates Hypothesis 1, positing that income inequality impedes Internet diffusion. These initial statistical observations offer preliminary evidence for further empirical exploration of the impact of income inequality on Internet diffusion. However, as the scatter plots and fitted lines are drawn without factoring in other variables, the results may be skewed. Thus, a more in-depth regression analysis is required to reach more robust conclusions.

### 4.2 Baseline regression results

Based on Equation 1, Table 2 presents the baseline regression results. Notably, the regression coefficients of *Ig* in column (1)–(4) are all significantly negative at the 1% level, regardless of the inclusion of control variables. The finding indicates that income inequality exerts a significantly negative impact on Internet diffusion. This conclusion aligns with the work of Gulati and Yates (2) as well as Billon et al. (7). Moreover, the empirical test results for the city sample align with those at the province sample, providing robust support for Hypothesis 1.

**Table 2:** The baseline regression results.

Variables	Model (1)	Model (2)	Model (3)	Model (4)
	Province sample		City sample	
	ln*Ius*	ln*Ius*	ln*Ius*	ln*Ius*
*Ig*	−1.957*** (0.735)	−2.412*** (0.932)	−0.886*** (0.075)	−0.821*** (0.074)
ln*Ent*		0.150** (0.071)		0.103*** (0.021)
*Mar*		−0.017 (0.038)		0.014** (0.007)
*Ps*		−5.750*** (1.763)		−0.002 (0.001)
ln*Roa*		0.592*** (0.202)		0.110*** (0.026)
ln*Iil*		0.353*** (0.117)		−0.030 (0.020)
ln*Une*		0.057 (0.111)		−0.064*** (0.020)
*Cri*		−0.164 (0.627)		0.004** (0.001)
fixed effect	YES	YES	YES	YES
sample size	480	480	4,352	4,352
*R* ^2^	0.791	0.813	0.893	0.895

*, ** and *** denote significant at 10, 5 and 1% significance levels, respectively; robust standard errors in parentheses.

In column (1), we initially consider only the fixed effects of provinces and years. The analysis reveals that a 0.1 unit increase in the regional Gini coefficient leads to a 19.57% decrease in the number of internet users per 100 people. In column (2), when control variables are incorporated, the magnitude of the effect slightly increases to 24.12%. Shifting our focus to the city level, in column (3), with only the fixed effects of cities and years considered, a 0.1 unit increase in the urban Gini coefficient results in an 8.86% decrease in the number of broadband internet users per 100 people. In column (4), upon including control variables, the inhibition effect slightly decreases to 8.21%.

A previous study by Ali et al. (45) concluded that income inequality promotes ICT diffusion among low-income groups in Australia, a view that starkly contrasts with our findings. The underlying reason for their result lies in the fact that the Internet has become a necessity in Australia. In contrast, China, as a large developing country experiencing rapid economic development and improving living standards, still has a significant number of low-income families in relative poverty. For these families, food, clothing, housing, and transportation remain the top priorities, while Internet products have not yet reached the status of necessities. In essence, the expansion of income inequality in China leads to a continuous increase in the size of low- and middle-income groups. Meanwhile, the consumption willingness and preference of these groups are predominantly focused on the necessities. Consequently, under such circumstances, the rate of Internet diffusion is substantially reduced. This finding clearly demonstrates that consumers’ willingness and motivation to consume Internet products are intricately linked to their economic status.

As for the results of control variables in Table 2, ln*Ent* has a significant positive impact on Internet diffusion. Current entrepreneurial activities in China are mainly concentrated in digital economy-related industries, such as live streaming with goods and the Netflix economy. Thus, these industries have a high degree of dependence on Internet. An increase in the entrepreneurship level will inevitably lead to a corresponding increase in Internet diffusion. The variable *Mar* has no significant effect on Internet diffusion at province sample, but significantly promotes Internet diffusion at city sample. The variable ln*Roa* has a significantly positive effect on Internet diffusion. The variable ln*Iil* has a significantly positive effect on Internet diffusion. The gap in network infrastructure between urban and rural areas in China is more obvious. Also, the geographical environment and living conditions in some regions, with their high construction costs, are not conducive to the network infrastructure construction. This leads to an insufficient network infrastructure and lack of information technology resources in less-developed regions, which makes it more difficult for residents to access information technology. The variable ln*Une* significantly inhibits Internet diffusion.

### 4.3 Robustness tests

#### 4.3.1 Replacement variables

##### 4.3.1.1 Replacement of the dependent variable

Internet diffusion rate mainly reflects the degree of Internet ownership, which refers to the difference in the information technology accessibility. The per capita telecommunication service volume reflects the degree of Internet application, which refers to the difference in the information technology use. For a robustness test, we introduce ln*Int* and *Ttv* to replace ln*Ius* as the dependent variable. The regression results are shown in Table 3. Individual and time fixed effects are controlled in all models. Control variables are only included in Models (2), (4), (6) and (8). The coefficient values of *Int* and *Ttv* are all significant negative, indicating that the baseline regression results are robust.

**Table 3:** Robustness test results with dependent variable replacement.

Variables	Model (1)	Model (2)	Model (3)	Model (4)
	Province sample		City sample	
	ln*Int*	*Ttv*	ln*Int*	*Ttv*
*Ig*	−2.944*** (0.946)	−0.584*** (0.188)	−0.771*** (0.070)	−0.177*** (0.035)
Control variables	YES	YES	YES	YES
Fixed effect	YES	YES	YES	YES
Sample size	480	480	4,352	4,352
*R* ^2^	0.886	0.966	0.941	0.620

*, ** and *** denote significant at 10, 5 and 1% significance levels, respectively; robust standard errors in parentheses.

We can find from column (1) that for every 0.1 unit increase in the provincial Gini coefficient, the total number of internet users decreases by 29.44%. In column (2), we can find that for every 0.1 unit increase in the provincial Gini coefficient, the per capita telecommunications volume decreases by 5.84%. We can find from column (3) that for every 0.1 unit increase in the prefecture-level Gini coefficient, the total number of internet users decreases by 7.71%. In column (4), the magnitude of the negative effect decreases to 1.77%.

##### 4.3.1.2 Replacement of the independent variable

Due to the lack of substitute indicators for independent variables in city sample, we use only province sample for robustness testing. Here, we measure the Gini coefficient using nighttime light data and per-capita GDP data of cities within each province. In the robustness test, the Gini coefficient serves as a proxy for income inequality. The regression results of columns (2) and (4) in Table 4 show that the coefficient values of *Ig_G* and *Ig_L* are significantly negative at the 1% level. This article examines the fixed effects of provinces and years in column (1). This indicates that income inequality negatively impacts Internet diffusion, thus validating the robustness of the baseline regression results.

**Table 4:** Robustness test results with independent variable replacement.

Variables	Model (1)	Model (2)	Model (3)	Model (4)
	ln*Ius*	ln*Ius*	ln*Ius*	ln*Ius*
*Ig_G*	−4.385***(0.897)	−4.963***(0.847)		
*Ig_L*			−1.783**(0.699)	−2.440***(0.740)
Control variables	NO	YES	NO	YES
Fixed effect	YES	YES	YES	YES
Sample size	416	416	416	416
*R* ^2^	0.831	0.851	0.816	0.834

*, ** and *** denote significant at 10, 5 and 1% significance levels, respectively; robust standard errors in parentheses.

#### 4.3.2 Controlling for endogeneity

In the baseline regression, we apply the ordinary least squares (OLS) method for estimation. Given that Internet diffusion is a dynamic process, its current period state not only depends on present-day factors but is also influenced by the previous-period diffusion level. Moreover, some independent variables affecting Internet diffusion can be reciprocally affected by it, indicating reverse causality. To address this, we introduce the first-order lagged term of Internet diffusion (*L_*ln*Ius*) as an independent variable into the model (45). Then, we use the system generalized moments (SYS-GMM) method for parameter estimation. Table 5 presents the SYS-GMM estimation results and relevant diagnostic test values. The P-statistic values of AR (2) are all above 0.1, failing to reject the null hypothesis, meaning no second-order autocorrelation exists. Meanwhile, the P-statistic values of the Hansen test are also greater than 0.1, validating the instrumental variables and the suitability of the SYS-GMM estimation method.

**Table 5:** SYS-GMM regression results.

Variables	Model (1)	Model (2)	Model (3)	Model (4)	Model (5)	Model (6)
	Province sample			City sample		
	ln*Ius*	ln*Int*	*Ttv*	ln*Ius*	ln*Int*	*Ttv*
*L_*ln*Ius*	0.675*** (0.020)			0.610*** (0.036)		
*L_* ln*Int*		0.732*** (0.047)			0.803*** (0.041)	
*L_Ttv*			1.068*** (0.147)			0.657*** (0.077)
*Ig*	−0.233*** (0.083)	−0.248*** (0.090)	−0.239*** (0.139)	−0.968*** (0.161)	−0.444*** (0.152)	−0.167*** (0.044)
Control variables	YES	YES	YES	YES	YES	YES
Fixed effect	YES	YES	YES	YES	YES	YES
Sample size	420	420	420	3,808	3,808	3,808
Number of instrumental variables	102	182	49	266	77	269
*F*	5549.23	1426.46	944.69	1069.72	1933.21	54.75
*AR* (1)	0.002	0.001	0.016	0.000	0.000	0.000
*AR* (2)	0.884	0.974	0.149	0.377	0.106	0.493
*Hansen test*	1.000	1.000	0.979	0.301	0.112	0.284

*, ** and *** denote significant at 10, 5 and 1% significance levels, respectively; robust standard errors in parentheses.

In Models (1) to (3), ln*Ius*, *Int*, and *Ttv* are set as dependent variables, respectively. The result of model (1) in Table 5 shows that the coefficient of *L_*ln*Ius* is significantly positive, indicating the continuity in the change of the Internet diffusion rate and the inertia effect of the previous-period rate on the current one. The coefficient values of *Ig* are all significantly negative at the 1% level, demonstrating that income inequality has a negative impact on Internet diffusion, further confirming the robustness of the baseline regression results. The regression results of models (2)–(6) exhibit similar patterns, further demonstrating the robustness of the baseline regression results.

#### 4.3.3 Instrumental variable methods

Selecting appropriate instrumental variables for the core explanatory variables can effectively address endogeneity issues and more accurately identify the causal relationship between the income inequalities and Internet diffusion. According to the method of Zhu and Lan (52), we use income inequality index from 2001 for cities as an instrumental variable representing the income inequality level. For panel data econometric analysis, we introduce a time-varying variable. Specifically, we construct an interaction term reflecting national income inequality in the previous year and income inequality index of cities in 2001. The income inequality in the city in 2001 (lagged variable) is correlated with the current income inequality in the city. Also, the influence of historical income inequality has diminished over time and does not directly impact recent social and economic development or Internet diffusion, thus satisfying the exclusivity condition.

Table 6 shows the empirical results of the instrumental variable methods. The results of Models (1) to (2), indicate that, after accounting for endogeneity, the regression coefficients of the impact of income inequality on Internet diffusion remain significant negative. This suggests that the inhibitory effect of income inequality on Internet diffusion is robust.

**Table 6:** Robustness test results for instrumental variables.

Variable	Model (1)	Model (2)
DE instrumental variable	−4.881*** (0.783)	−5.261*** (0.924)
Control variable	No	Yes
Fixed effect	Yes	Yes
Constant term	3.916*** (0.340)	2.622*** (0.547)
KPL	34.013***	27.815***
Cragg-Donald Wald Value	107.409 > 16.380	86.082 > 16.380
Kleibergen-Paap rk Wald Value	28.879 > 16.380	23.928 > 16.380
Sample size	4,352	4,352
R^2^ value	0.797	0.779

*, **, and ***, respectively, indicate significance at the 10, 5, and 1% levels, with robust standard errors in parentheses.

Additionally, the under-identification test shows that the *p*-values of the KPL (Kleibergen-Paap rk LM) statistic for the instrumental variable is less than 0.01. This significant p-value rejects the null hypothesis of under-identification, indicating that the instrumental variable is reasonable and valid.

In the weak instrumental variables test, the Wald F statistic for the instrumental variable, based on the Cragg-Donald and Kleibergen-Paap rk criteria, exceeds the critical value at the 10% level of the Stock-Yogo weak identification test. This confirms that the instrumental variable meets the correlation requirement. Overall, these tests demonstrate that the instrumental variable is reasonable.

#### 4.3.4 Sensitivity analysis

Income inequality may be potentially affected by urban development level and other factors, and the difference in urban development level is also an important reason for the Internet diffusion in cities. To obtain a consistent estimate of the true coefficient α_1_ of regional income inequality, it is necessary to eliminate the impact of the correlation between income inequality and urban development level on model estimation. We collected a large number of control variables and introduced fixed effects of time and region to control for unobservable factors. However, we cannot incorporate all control variables into the model, and inevitably face endogeneity issues caused by omitted variables. To alleviate macro systemic environmental changes, we introduce the interaction effect between provinces and years in the benchmark model. The regression coefficient of the independent variable Ig is −0.511, which is significantly negative at the 1% level, and the R^2^ is 0.926, indicating that the regression results in the previous section are robust.

At the same time, this article draws on Oster’s (53) method for coefficient sensitivity analysis to examine the impact of potential omitted variables on regression estimation results. The Oster method uses the correlation information between observable variables and independent variable to estimate the correlation between unobservable variables and the independent variable, in order to estimate the magnitude of bias caused by omitted variables. Oster provided two methods for testing whether omitted variables would affect empirical results (the command in Stata is psacalc). This method requires setting two key parameters: *δ* and R_max_. And, *δ* represents the selection proportionality, which is used to measure the strength of the correlation between omitted variables and the dependent variable compared to the correlation between the observable variables and the dependent variable. R_max_ represents the maximum goodness of fit of the regression equation if unobservable omitted variables can be observed. Method 1: Given a δ (usually set to 1) and a R_max_, the coefficient estimate α_1_* of the independent variable is simulated to form the range of values for the variable estimation coefficient. When the range of values does not include 0, a robustness test is passed. Method 2: Given a R_max_ that includes omitted variables, and assuming the estimated coefficient α_1_ of the independent variable is 0, calculate *δ*; If δ > 1, it indicates that the problem of missing variables is not serious.

In terms of specific operations, we set the R_max_ in the Oster omitted variable test to 0.940 (the R_max_ increased by 5% compared to the R^2^ of the benchmark regression, which is greater than the R^2^ obtained by the province year interaction effect model, indicating that more omitted variables were considered) for robustness testing. The results are shown in Table 7. Among them, the first line is the range of values composed of α_1_* obtained after setting δ = 1, which does not include 0 and has passed the robustness test. The second line is the estimated value of δ obtained when setting α_1_ = 0, and the result shows that δ is greater than 1, indicating that missing variables make it difficult to change the significance of the benchmark results.

**Table 7:** Coefficient sensitivity test.

Method	Criteria	Calculation results	Passed or not
Method one	The value range does not include 0	[−0.821,-0.335]	Passed
Method two	|δ(α_1_ = 0, R_max_ = 0.940)| > 1	δ = 1.163	Passed

### 4.4 Threshold regression results

To verify the nonlinear effect of income inequality on Internet diffusion, we use the threshold panel model for the empirical test. This model suits large data samples better. Given that the small sample size of provincial panel data might lead to inaccurate threshold tests, our analysis of the nonlinear effect is based on prefecture-level city panel data. We use *Ig* as the core independent variable, and *Ig*, ln*GDP*, *Huc*, and *Soc* as the threshold variables. Prior to estimating with the threshold model, a panel threshold existence test was performed following the method of (56). Using the bootstrap method, 1,000 repeated samplings were carried out. The test results are presented in Table 8. The results reveal that the threshold variable *Ig* does not pass the threshold test when *Ig* and other variables are used as core independent variables. The three threshold variables ln*GDP*, *Huc*, and *Soc* pass the single and double threshold tests but not pass the triple threshold test.

**Table 8:** Threshold effect test results of city sample.

Independent variables	Threshold variables	threshold	*F*	*p*	1% threshold	5% threshold	10% threshold
*Ig*	*Ig*	single threshold	24.35	0.1330	40.7650	31.2904	26.2345
		double threshold	21.14	0.1470	50.4235	32.0277	25.4045
		triple threshold	15.32	0.4270	48.7422	36.5821	28.5029
*Ig*	ln*GDP*	single threshold	437.55***	0.0000	40.0557	30.2506	25.5307
		double threshold	186.34***	0.0000	37.2953	26.6417	22.9391
		triple threshold	80.96	0.6450	207.8965	180.9668	167.4812
*Ig*	*Huc*	single threshold	247.04***	0.0000	47.9842	32.2766	27.6000
		double threshold	111.70***	0.0000	38.9139	28.2946	24.0630
		triple threshold	19.88	0.5730	64.8060	47.3549	41.1572
*Ig*	*Soc*	single threshold	437.42***	0.0000	36.9091	27.5392	22.8617
		double threshold	142.94***	0.0000	35.0592	24.6014	20.5721
		triple threshold	73.51	0.5380	144.3692	120.7860	109.6783

*, ** and *** denote significant at 10, 5 and 1% significance levels, respectively.

Based on Equation 2, the threshold regression results are shown in Table 9. In Model (1), when ln*GDP* breaks through the threshold *q*1, the coefficient of *Ig* decreases sharply, from −1.182 to −0.649. When ln*GDP* is further increased to break through the threshold *q*2, the coefficient of *Ig* further decreases from −0.649 to −0.338, and is significant at the 1% level. This finding indicates that, when ln*GDP* rises, the impact of income inequality on Internet diffusion continues to weaken. The results of Models (2) and (3) show that the impact of income inequality on Internet diffusion continues to weaken as the values of *Huc* and *Soc* increase. Therefore, the negative impact of income inequality on Internet diffusion can be mitigated by continuously increasing the level of human and social capital.

**Table 9:** Threshold regression results.

Variables		Model (1)	Model (2)	Model (3)
Threshold variables		ln*GDP*	*Huc*	*Soc*
Estimated threshold	*q*1	0.2580	4.9759	3.3190
	*q2*	1.0240	6.3954	3.9439
*Ig*×*I*(*Th*≤*q*1)		−1.182*** (0.113)	−1.271*** (0.124)	−1.384*** (0.125)
*Ig*×*I*(*q*1 < *Th*<*q*2)		−0.649*** (0.101)	−0.800*** (0.115)	−0.959*** (0.110)
*Ig*×*I*(*Th*≥*q*2)		−0.338*** (0.100)	−0.552*** (0.110)	−0.538*** (0.101)
Control variables		YES	YES	YES
Fixed effect		YES	YES	YES
Sample size		4,352	4,352	4,352
*R* ^2^		0.878	0.871	0.877

*, ** and *** denote significant at 10, 5 and 1% significance levels, respectively; robust standard errors in parentheses.

By comparison, the improvement of economic development level can most weaken the inhibitory effect of income inequality on Internet diffusion. Within the sample range, the weakening effect is strongest when the per capita GDP of the city exceeds 27,800 yuan, but the impact coefficient of *Ig*×*I* (*Th*≥*q*2) still has −0.338 and is significantly negative at the 1% level. This conclusion is consistent with the findings of Beilock and Dimitrova (1), Billon et al. (7), Ali et al. (45) as well as Gulati and Yates (2), indicating that the influence of income on Internet penetration rate diminishes as income levels rise. The results indicate that while the growth of economic development, human capital, and social capital can mitigate the inhibitory impact of regional income inequality on Internet diffusion, they cannot eliminate it.

### 4.5 Heterogeneity analysis

The process of Internet diffusion in a geographical area is jointly determined by users’ individual decisions and enterprises’ choices to offer Internet services in specific locations. From the demand side, Internet users’ decisions hinge on their willingness to pay for Internet technology. From the supply side, relevant enterprises’ investment decisions are shaped by their profit-making ability and potential in the Internet market. There are substantial differences in economic development, population structure, and network infrastructure between eastern China and central and western China. Income inequality also impacts Internet diffusion differently across regions (4), an aspect that requires in-depth exploration. We classify China’s prefecture-level cities into eastern cities and mid-western cities, following the criteria in the China Health Statistical Yearbook.^2^

Furthermore, the level of urban innovation exerts a profound influence on lowering Internet access costs, promoting knowledge spillovers, and enhancing its efficiency in converting digital dividends into tangible benefits (35, 51). Therefore, we also examine the differences of the impact of the regional income inequality on Internet diffusion between innovative and non-innovative cities. This classification framework is anchored in a landmark national policy initiative. In 2005, the State Council of China issued *the Outline of the National Medium- and Long-Term Program for Science and Technology Development (2006–2020)*, which enshrined the establishment of an innovation-driven nation as a landmark long-term strategy. Subsequently, China formally initiated the drive to develop innovative cities. By the end of 2022, the National Development and Reform Commission (NDRC) and the Ministry of Science and Technology (MOST) had cumulatively approved 101 national innovative pilot cities over seven batches.

In the selection of these innovative pilot cities, the state undertakes a comprehensive evaluation of multifaceted factors, aimed at identifying cities with potential for innovation-driven growth and demonstrative effects. This selection process is designed to explore innovation-driven development pathways and develop replicable experiential models. Core factors under evaluation include the city’s foundational innovation conditions, intensity of innovation investment, performance of innovation outputs, and support for the innovation ecosystem. Therefore, these pilot cities generally exhibit a relatively high level of innovation leadership across the country. Consequently, national innovative pilot cities are defined as the innovative city sample, whereas all other cities constitute the non-innovative city sample. Due to constraints on variable data availability, the final study sample includes a total of 94 national innovative pilot cities.

First, it conducts a descriptive statistical analysis of the disparities in Internet diffusion and income inequality between these two groups (Table 10). Evidently, Internet consumption is higher in the eastern and innovative cities, while their income inequality level is lower. This finding indicates the necessity of a heterogeneity test.

**Table 10:** Differences in Internet diffusion and Income Inequality.

Variables	Eastern cities average	mid-western cities average	Difference in means between groups	Innovative cities average	Non-innovative cities average	Difference in means between groups
ln*Ius*	2.724	2.258	0.466***	2.770	2.247	0.522***
*Ig*	0.454	0.630	−0.177***	0.483	0.610	−0.127***

*, ** and *** denote significant at 10, 5 and 1% significance levels, respectively.

The results of heterogeneity test are shown in Table 11. The coefficients of *Ig* are significantly negative at the 1% level, both across eastern and mid-western regions, and between innovative and non-innovative cities. However, compared with the eastern and innovative cities, the absolute coefficient value of *Ig* is higher in the mid-western and non-innovative cities. This finding indicates that regional and innovation differences exist in the inhibitory effect of income inequality on Internet diffusion. In addition, the effects on Internet diffusion in the mid-western and cities are stronger than in the eastern and innovative cities.

**Table 11:** Results of heterogeneity analysis.

Variables	(1)	(2)	(3)	(4)
	Eastern cities	Mid-western cities	Innovative cities	Non-innovative
	ln*Ius*	ln*Ius*	ln*Ius*	ln*Ius*
*Ig*	−0.429***(0.133)	−0.731*** (0.085)	−0.471*** (0.149)	−0.699*** (0.078)
Control variables	YES	YES	YES	YES
Fixed effect	YES	YES	YES	YES
Sample size	1,584	2,768	1,504	2,848
*R* ^2^	0.882	0.900	0.866	0.910

*, ** and *** denote significant at 10, 5 and 1% significance levels, respectively; robust standard errors in parentheses.

According to Beilock and Dimitrova (1), income have a more substantial effect on Internet penetration during low-income stages. Therefore, the regional heterogeneity results is primarily attributed to the combined effects of differences in economic foundations, industrial structures, and social security systems between the eastern and mid-western regions of China. Residents in the mid-western parts of the country experience more salient constraints on their Internet-related consumption due to lower income levels, lagging infrastructure, and less comprehensive social security systems (36, 46). The consumption structure of these residents places a higher proportion of expenditure on daily necessities, whereas Internet-related consumption is generally regarded as non-essential goods and services (18, 29). Furthermore, the lower levels of social protection systems in these regions exacerbate the sense of future uncertainty among the population, leading to a stronger motivation for precautionary savings (38). This directly suppresses various types of consumption, including Internet-related consumption.

At the same time, internet technological innovation can continuously improve the speed and efficiency of Internet access while simultaneously reducing the costs of Internet use, thereby promoting Internet diffusion (10). The innovative cities benefits from a well-developed digital infrastructure, which significantly reduce the transaction costs and accessibility barriers associated with Internet-related consumption (32). Consequently, even lower-income groups within this region find it easier to participate in Internet use, which partially mitigates the limitations imposed by their income constraints.

## 5 Mechanism analysis

### 5.1 Model settings

In order to further test the impact mechanism of income inequality on Internet diffusion, we construct the following models:


(3) $$\mathrm{Me}d_{i,t}=\gamma_{0}+\gamma_{1}Ig_{i,t}+\lambda_{j}\sum_{j-1}^{J}X_{i,j,t}+u_{i}+v_{t}+\varepsilon_{i,t}$$

(4) $$\ln Ius_{i,t}=\delta_{0}+\delta_{1}Ig_{i,t}+\delta_{2}\mathrm{Me}d_{i,t}+\lambda_{j}\sum_{j-1}^{J}X_{i,j,t}+u_{i}+v_{t}+\varepsilon_{i,t}$$

Where *Med_i,t_* denotes the mediating variables, including the three mediating variables of economic development level (ln*GDP*), human capital level (*Huc*), and social capital level (*Soc*). The remaining variables are the same as in Equation 1.

### 5.2 Mechanism test results

We first test the economic suppressive effect proposed in Section 2.2, based on Equations 3 and 4, the regression results are shown in Table 12. In Column (1) of Table 12, the regression coefficient of *Ig* is significantly negative at the 1% level, indicating that income inequality has a significantly negative effect on economic development. Subsequently, the variable ln*GDP* is incorporated into the regression model, with judgment based on observing the coefficient value change and the significance of *Ig*. In Column (2) of Table 2, the coefficient of *Ig* is −2.412, significant at the 1% level. In Column (2) of Table 12, the coefficient of *Ig* is −1.963, significant at the 5% level. Compared with the baseline results in Table 2, both the coefficient value and significance of *Ig* have decreased substantially, indicating that economic development is one of the mediating mechanisms. Thus, Hypothesis 2 is verified. In Columns (3) to (4), the results for the city sample are consistent with the province sample. The results also suggest that income inequality will hinder economic development, which in turn will hinder infrastructure construction, especially Internet infrastructure.

**Table 12:** The results of economic suppressive effect.

Variables	Model (1)	Model (2)	Model (3)	Model (4)
	Province sample		City sample	
	ln*GDP*	ln*Ius*	ln*GDP*	ln*Ius*
*Ig*	−0.926*** (0.256)	−1.963** (0.957)	−0.430*** (0.036)	−0.515*** (0.070)
ln*GDP*		0.484** (0.239)		0.710*** (0.032)
Control variables	YES	YES	YES	YES
Fixed effect	YES	YES	YES	YES
Sample size	480	480	4,352	4,352
*R* ^2^	0.980	0.815	0.964	0.907

*, ** and *** denote significant at 10, 5 and 1% significance levels, respectively; robust standard errors in parentheses.

We further test the education crowding-out effect. In Column (1) of Table 13, the coefficient of *Ig* is significantly negative at the 1% level, suggesting that income inequality has a significantly negative effect on human capital. The coefficient of *Ig* in Column (2) of Table 13 is −2.024 and significant at the 5% level. Compared with the baseline results, both the coefficient value and significance of *Ig* are decreased, indicating that human capital is a mediating mechanism, which is consistent with Hypothesis 2. In Columns (3) and (4), the results for the city sample are consistent with the province sample. The results suggest that a higher education level and better human capital can significantly accelerate Internet diffusion. This conclusion aligns with the findings of Pick and Nishida (51). Highly-educated residents are more inclined to seek Internet products and services than those with lower education levels (2, 47). Internet technology requires certain skills. Therefore, those with more knowledge have stronger Internet-using capabilities and can make greater use of the Internet, especially to gain more economic benefits (23). The Internet often holds little appeal for less-educated groups as they struggle with the complexities of Internet applications (34).

**Table 13:** Test results of class solidification effect and education crowding out effect.

Variables	Model (1)	Model (2)	Model (3)	Model (4)	Model (5)	Model (6)	Model (7)	Model (8)
	Province sample		City sample		Province sample		City sample	
	*Huc*	ln*Ius*	*Huc*	ln*Ius*	*Soc*	ln*Ius*	*Soc*	ln*Ius*
*Ig*	−1.754*** (0.427)	−2.024** (0.905)	−0.525*** (0.149)	−0.788*** (0.073)	−12.612*** (2.265)	−1.778* (0.925)	−1.349*** (0.197)	−0.634*** (0.065)
*Huc*		0.221** (0.110)		0.063*** (0.008)				
*Soc*						0.050 (0.032)		0.651*** (0.033)
Control variables	YES	YES	YES	YES	YES	YES	YES	YES
Fixed effect	YES	YES	YES	YES	YES	YES	YES	YES
Sample size	480	480	4,352	4,352	480	480	4,352	4,352
*R* ^2^	0.981	0.815	0.814	0.897	0.855	0.815	0.836	0.909

*, ** and *** denote significant at 10, 5 and 1% significance levels, respectively; robust standard errors in parentheses.

Finally, we test the class solidification effect. In Column (5) of Table 13, the coefficient of *Ig* is significantly negative at the 1% level, indicating that income inequality has a significantly negative effect on social capital. The regression coefficient of *Ig* in column (6) is −1.778 significant at the 10% level. Compared with the baseline results, both the coefficient value and significance of *Ig* are decreased, indicating that social capital is a mediating mechanism. This finding supports Hypothesis 2. In Columns (7) to (8), the results for the city sample are consistent with the province sample. Income inequality exacerbates network segregation by widening socioeconomic disparities, which creates communication barriers between different social groups. This segregation manifests primarily as a tendency toward homophily in social interactions; individuals are more inclined to establish connections with others of similar economic backgrounds and social status. As a result, low-income groups often remain confined within social networks composed predominantly of other low-income individuals, while high-income groups tend to interact within their own homogeneous circles (6). Network structures formed by resource-advantaged groups often exhibit a self-reinforcing effect: they enjoy greater access to the internet and acquire digital skills more rapidly, thereby further consolidating their privileged position. Conversely, resource-disadvantaged groups are often subject to negative network effects, characterized by low information accessibility, weaker internet proficiency, and potentially reinforced inertia. Such divergence is likely to lead to a self-perpetuating cycle: it restricts rational mobility across social strata and may even intensify class solidification, perpetuating structures of inequality across generations (13).

## 6 Conclusions and policy implications

### 6.1 Conclusion

China, home to over one-third of the world’s Internet users, is in the throes of an information and digital revolution. Meanwhile, China has well-defined strategies for poverty alleviation, building a moderately prosperous society, and achieving all round common prosperity. However, existing income inequality remains a “hurdle” in promoting Internet diffusion and common prosperity for all. While the impact of income inequality on consumption is acknowledged, further clarifying its effect on Internet diffusion is crucial for bridging the digital divide and driving the digital economy’s high-quality development. This paper empirically explores how income inequality impacts Internet diffusion. The analysis is based on effectively measuring income inequality via nightly lighting data and the Gini coefficient at provincial and municipal levels in China from 2005 to 2020. The aim is to offer decision making insights for China to accelerate the digital economy’s high-quality development by identifying the influence of income inequality on Internet diffusion.

The results indicate that income inequality significantly dampens Internet diffusion. This conclusion remains valid after replacing independent and core independent variables, and conducting robustness tests like the systematic GMM approach. As income inequality rises, its impact on Internet diffusion does not exhibit significant non-linear traits. However, when economic development, human capital, and social capital reach a certain level, the negative impact of income inequality on Internet diffusion weakens. Regarding regional heterogeneity, income inequality has a far more pronounced inhibitory effect on Internet diffusion in the mid-western and non-innovative cities than in the eastern and innovative cities. In terms of the intermediate mechanism, income inequality hinders the diffusion of the Internet by suppressing economic growth, constraining the development of education, and reinforcing the consolidation of social classes.

This paper focuses on the impact of income inequality on Internet diffusion, serving as a crucial addition to the research field on the impact of income inequality on consumption. Meanwhile, this paper identifies three significant paths through which income inequality inhibits Internet diffusion, laying an important literature foundation for subsequent research. Moreover, the research conclusions of this paper provide important references for the government to formulate consumption policies and income distribution policies.

### 6.2 Limitations and further studies

This study’s scope is limited to a macro-analysis of Internet diffusion, focusing on penetration and traffic metrics without exploring usage intensity, consumption types, or the quantity of services consumed. Furthermore, our analysis does not fully dissect variations across income groups, urban–rural divides, and regions (57), nor does it employ micro-level data. Building on this, future work should investigate these specific dimensions by segmenting user groups (e.g., youth vs. older adult) and utilizing micro-data from households, firms, or individuals to uncover the precise mechanisms linking income inequality to digital diffusion.

### 6.3 Policy impactions

The results of our paper have significant policy implications. First, considering that the baseline results of this paper is that income inequality inhibits the Internet diffusion, in order to achieve Internet diffusion further deeply and continuously narrow the digital divide, it is recommended to continuously reform in the income distribution, tax, and household registration systems should be further expedited. This will help continuously narrow the income, regional, and urban–rural gaps, removing obstacles to Internet diffusion promotion and the digital divide elimination, while fueling the high-quality development of the digital economy. Initially, efforts must be made to boost citizens’ income levels and refine the income distribution mechanism. This promotes equal opportunity and steadily expands the middle-income group, essentially eradicating the negative impact of income inequality on Internet diffusion and the digital divide. Moreover, efforts should be directed towards intensifying social security and tax relief policies for low- and middle-income families, and providing more transfer payment protection to low-income disadvantaged groups. By continuously enhancing the living standards of urban and rural residents, conditions conducive to Internet diffusion and the high-quality development of the digital economy can be created.

Second, given the regional heterogeneity in the impact of income inequality on Internet diffusion, which is closely tied to regional economic development levels, income distribution reform should be tailored to local conditions. Public livelihood inputs should be tilted more towards remote and underdeveloped areas in central and western China, especially in education, healthcare, and housing security (7). The goals are to continuously improve residents’ living standards, enhance local economic development by attracting talent and investment, mitigate the negative impact of income inequality on Internet diffusion, and further increase residents’ income through job creation. This creates a virtuous cycle. Moreover, increasing Internet diffusion in backward areas can encourage residents to start businesses or engage in activities like live-streaming and rural e-commerce, capitalizing on the low-threshold nature of the Internet economy for active innovation and entrepreneurship. New “Internet +” industries, paths, and models should be explored to promote common prosperity.

Third, considering that the mechanism analysis in this paper reveals that income inequality has a negative impact on Internet diffusion by suppressing human capital accumulation, therefore the government and the education sector should collaborate to establish and enhance the local human capital cultivation system. Local governments should step up efforts to assist the flexibly employed and Internet entrepreneurs, providing them with training in Internet and computer knowledge to improve their Internet-using skills. Meanwhile, local governments should actively guide Internet users to focus more on value-added Internet functions, such as online job-hunting, e-commerce, and Internet-based entrepreneurship. For those with entrepreneurial aspirations, organized learning and training are crucial for cultivating their Internet-using skills and literacy. Additionally, they should leverage Internet platforms to expand their social networks and boost social capital. With the continuous advancement and application of emerging technologies (such as AI, big data, cloud computing, and IoT) and the growing integration of the digital and real economies, new business models and jobs have emerged. Thus, the education sector, especially colleges and universities, should strengthen the cultivation of Internet-technology professionals and deepen industry-education integration.

Finally, policy guidance should be fully utilized to boost Internet speeds and cut costs. Local economic development is pivotal in achieving these goals. As we have verified, income inequality’s economic inhibition hamper local Internet diffusion. Upgrading information infrastructure in scale, quality, and efficiency can also boost Internet consumption. Government should guide and encourage Internet enterprises to invest more in Internet infrastructure in backward and rural areas. For instance, China’s “East Data and West Computing” project addresses the resource imbalance between east and west by building large-scale data center computing facilities in the west for the east’s high computing demand. To tackle common rural Internet issues (weak signals, poor infrastructure, slow speeds, high costs), telecom companies should be spurred to conduct high-quality network construction like 5G and gigabit fiber in rural areas. Provide financial subsidies, subsidize low-income Internet users and SMEs to speed up rural Internet and cut costs, thus promoting Internet diffusion, and ensuring the digital economy’s benefits reach all citizens more equitably (54).

## Data Availability

The raw data supporting the conclusions of this article will be made available by the authors, without undue reservation.

## Footnotes

^1^Source: 53rd Statistical Report on the Development of the Internet in China.

^2^The eastern region includes Beijing, Tianjin, Hebei, Liaoning, Shanghai, Jiangsu, Zhejiang, Fujian, Shandong, Guangdong, and Hainan. The central and western region includes Heilongjiang, Jilin, Shanxi, Anhui, Jiangxi, Henan, Hubei, Hunan, Inner Mongolia, Guangxi, Chongqing, Sichuan, Guizhou, Yunnan, Tibet, Shaanxi, Gansu, Qinghai, Ningxia, and Xinjiang.
